# In-situ neutron diffraction study of lattice deformation behaviour of commercially pure titanium at cryogenic temperature

**DOI:** 10.1038/s41598-022-07640-3

**Published:** 2022-03-08

**Authors:** Min-Su Lee, Takuro Kawasaki, Takayuki Yamashita, Stefanus Harjo, Yong-Taek Hyun, Youngung Jeong, Tea-Sung Jun

**Affiliations:** 1 Department of Mechanical Engineering, Incheon National University, Incheon, 22012 Republic of Korea; 2 J-PARC Center, Japan Atomic Energy Agency, Tokai, Ibaraki 319-1195 Japan; 3 Metallic Materials Division, Korea Institute of Materials Science, Changwon, 51508 Republic of Korea; 4 School of Materials Science and Engineering, Changwon National University, Changwon, 51140 Republic of Korea; 5 Research Institute for Engineering and Technology, Incheon National University, Incheon, 22012 Republic of Korea

**Keywords:** Engineering, Materials science

## Abstract

Titanium has a significant potential for the cryogenic industrial fields such as aerospace and liquefied gas storage and transportation due to its excellent low temperature properties. To develop and advance the technologies in cryogenic industries, it is required to fully understand the underlying deformation mechanisms of Ti under the extreme cryogenic environment. Here, we report a study of the lattice behaviour in grain families of Grade 2 CP-Ti during *in-situ* neutron diffraction test in tension at temperatures of 15–298 K. Combined with the neutron diffraction intensity analysis, EBSD measurements revealed that the twinning activity was more active at lower temperature, and the behaviour was complicated with decreasing temperature. The deviation of linearity in the lattice strains was caused by the load-redistribution between plastically soft and hard grain families, resulting in the three-stage hardening behaviour. The lattice strain behaviour further deviated from linearity with decreasing temperature, leading to the transition of plastically soft-to-hard or hard-to-soft characteristic of particular grain families at cryogenic temperature. The improvement of ductility can be attributed to the increased twinning activity and a significant change of lattice deformation behaviour at cryogenic temperature.

## Introduction

Titanium and its alloys have excellent mechanical properties, corrosion resistance and low-temperature performance^1–3^. Most of metallic materials exhibit a ductile-to-brittle transition at low temperature, whilst several Ti alloys with a low impurity show better structural material properties (e.g., fatigue and toughness)^3–5^, and surprisingly higher ductility at cryogenic temperature^4,6,7^. This signifies a potential of improving the formability of Ti through cryogenic processing techniques (e.g., cryo-rolling and -forging)^8–11^. Due to the promising low-temperature properties, much attentions have been paid to its cryogenic applications such as liquid rocket engine, cryogenic fuel storage tank and transportation of liquid gas from offshore^2,5,12^. In addition, there are efforts to develop nanocrystalline Ti with a superior performance using cryogenic processing, in which the grain size is further refined due to the increase of twinning activity with decreasing temperature^8–11^. The lattice orientation in original grain matrix is rotated by twinning, and in turn, creating a new interface (i.e., twin boundary). Each twinning systems has a particular misorientation (θ), and the twinning-induced lattice reorientation contributes to the texture evolution based on the twin boundary misorientation angle^13,14^. The twin boundary can act as either an obstacle or pathway for dislocation glide depending on its interfacial characteristics^15^. In α-titanium, the $\left\{ {10\overline{1}2} \right\}$ extension twin (θ = 85°) and $\left\{ {11\overline{2}2} \right\}$ contraction twin (θ = 64.4°) are two common twinning systems at room temperature. The activation of twinning systems depends on the crystal orientations to the applied stress condition^14^, and the unusual type of twin has been occasionally reported at extreme deformation conditions, i.e., fast strain rate^16,17^ and cryogenic temperature^18^. Therefore, it is worthy to attempt the various processing routes to control twinning with taking into account the crystallographic texture at cryogenic temperature.

In addition to deformation twinning, dislocation slip also acts as a carrier of plasticity at both room and cryogenic temperature (e.g., 77 K)^18,19^. Previous studies showed in pure Ti that the dislocations were pinned at interstitial impurities (especially oxygen solutes), resulting in a strong repulsion in short range strain field between the defect structures^20^. The pinned dislocation can bypass the microstructural barrier through the atomic shuffling^18^ and dislocation cross-slip or climb^18,21^. This dislocation-pinning and -unpinning process would be a crucial mechanism for strain-hardening together with twinning-induced hardening mechanism, and the dislocation pinning effect is further prominent in Ti alloys with a low impurity due to the absence of alloying compounds and heterogeneous phase structures. At room temperature, the dislocation slip behaviour was examined in details of slip systems for α-Ti; in general, < a > -dislocation slip on prismatic (*Pri* < a >) and basal plane (*Ba* < a >), and < c + a > -pyramidal slip on first order plane (*Pyr* < c + a >). The *Pri* < a > and *Ba* < a > slip exhibited planar and wavy slip behaviours^21–23^, respectively. The < a > -dislocation glide on prismatic plane showed a planar-to-wavy slip transition at room-to-cryogenic temperature^18,19^, and the wavy slip is involved in the cross-slip process^18,21,23^. Thus, it is suggested that the nature of slip systems exerts an impact on the plastic behaviour of polycrystalline titanium; for example, in lack of twinning deformation, the strain-hardening behaviour was distinct between two texture conditions with different active slip systems^6,24,25^. The activation of deformation modes (i.e., slip and twinning) strongly depends on two material-based factors: (i) crystallographic orientations (i.e., texture) and (ii) critical revolved shear stress (CRSS). As temperature decreases, the CRSS for dislocation slip is rapidly enhanced due to the increase of lattice friction, promoting deformation twinning^7,26^. The CRSS for slip systems of α-Ti has been evaluated using a micro-mechanical testing at room temperature, and it is rather clear that the ratio of CRSS is ~ 3:4:9 in order of *Pri* < a > , *Ba* < a > and *Pyr* < c + a > slip systems^22^. However, in low temperature range, the relevant CRSS was only estimated by the macroscopic experiments using bulk samples with microstructural uncertainty^27–29^, so it is rather ambiguous. Besides the detail of Pyr < c + a > slip is absent at temperatures lower than room temperature. The activity of slip systems plays an important role in the mechanical properties of Ti and this could be different at cryogenic temperature. Nevertheless, the activation of slip systems has not been explored at cryogenic temperature. Fundamental understanding of the effect of (cryogenic) temperature on intrinsic material defects in Ti will contribute to the advance of industrial fields at extreme cryogenic environments.

Since the plasticity of well-oriented grains in polycrystalline α-Ti initiates with the activation of the preferred slip systems, which satisfy their CRSS in the given orientation and applied stress conditions, the local deformation response is highly sensitive to the lattice orientation^30–33^. *In-situ* time-of-flight neutron diffraction is a powerful tool to study the deformation mechanisms of a statistically large number of grains due to the deep penetration capability of neutron beams, thus providing a reliable data set from the bulk polycrystalline samples^34,35^. The time-of-flight technique permits the wavelength-resolved diffraction measurements over the broad range of wavelengths^36^. The refined crystallographic information can be extracted by the analysis of the neutron spectra, regarding to the reflection-dependent lattice spacings, grain orientation and phase volume fraction. This *in-situ* technique enables to acquire the diffraction data during the testing process, whereby the deformation responses of constituent microstructures can be monitored in both elastic and plastic regime, especially at the elastic-to-plastic transition Section. ^30–33^.

In the present study, *in-situ* neutron diffraction tensile tests were performed to explore the lattice behaviour of α-Ti in certain grains with similar crystal orientation at temperatures of 298 K, 123 K and 15 K. We found a significant change of lattice strain evolution with the transition of plastically soft-to-hard and hard-to-soft characteristic in certain grain families at cryogenic temperature, probably suggesting that a particular slip system can be promoted with decreasing temperature. The superior cryogenic properties of α-Ti were documented by the lattice deformation related to the dislocation slip and increased twinning activity.

## Materials and methods

**Figure 1:**
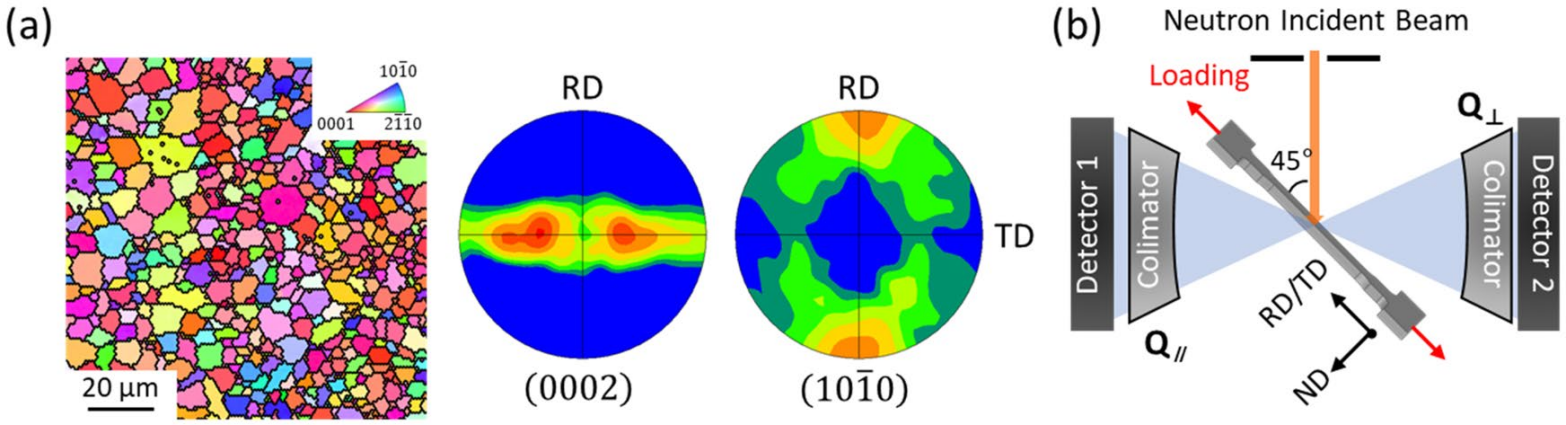
(**a**) EBSD IPF map, and ($0002$) and ($10\overline{1}0$) pole figures of as-received Grade 2 CP-Ti plate. (**b**) A schematic illustration of in-situ neutron experimental setup.

A rolled Grade 2 commercially pure titanium (CP-Ti) plate was used in this study. The microstructure and crystallographic texture of the as-received material was characterised by electron backscatter diffraction (EBSD) technique, as shown in Fig. 1a. The equiaxed grain structure with a mean grain size of 4.2 μm (in diameter) is observed in the inverse pole figure (IPF) map. The ($0002$) pole figure exhibits a typical rolling texture of α-Ti^37^, in which the basal poles of hcp lattice structures are mostly concentrated at angels of ~ 35–40° away from the normal direction (ND) toward the transverse direction (TD) in rolled plane, and the remainders are widely distributed along the TD axis. The tensile samples were cut parallel and transverse to the rolling direction (RD) of CP-Ti plate.

*In-situ* time-of-flight neutron diffraction measurements were performed during the tensile tests using the TAKUMI (BL19) diffractometer at the Materials and Life Science Research Facility of Japan Proton Accelerator Research Complex^38,39^. The tensile samples were set on the loading machine horizontally. The loading axis (RD or TD) was oriented at + 45° relative to the incident neutron beam, and two detector banks with 5 mm collimators were located at ± 90° to collect the diffraction patterns with scattering vectors parallel to the loading direction (Q_||_) and the normal direction in rolled plane (${\text{Q}}_{ \bot }$), as illustrated in Fig. 1b. The former diffraction patterns (Q_||_) were only used in this work, and the latter diffraction patterns (${\text{Q}}_{ \bot }$) are presented in supplementary Fig. S1. The incident slit was 5 mm wide and 5 mm high. The tensile tests were conducted with a strain rate of 2 × 10^–5^ s^-1^ at 298 K, 123 K and 15 K. Full pattern fitting of the diffraction spectra was carried out by Rietveld refinement using the Z-Rietveld software^40^. The details of goodness-of-fit are described in the supplementary material. The lattice strains pertaining to {hkil} diffraction plane is defined as follows:

1 $$\varepsilon_{hkil} = \frac{{d_{hkil} - d_{0} }}{{d_{0} }}$$

where, $\varepsilon_{hkil}$ denotes the lattice strain; $d_{0}$ and $d_{hkil}$ are the interplanar lattice spacing of {hkil} plane at the stress-free and the loaded state, respectively.

The fractured tensile samples were obtained after the tensile tests at 298 K, 123 K and 15 K under RD and TD loading conditions. The regions below ~ 1 mm from the fracture surfaces of samples were cross-sectioned to examine the deformed microstructures. The cross-sectional area was mechanically ground with a series of SiC papers up to 4000 grit, and subsequently electro-polished in a solution of 410 ml methanol + 245 ml 2-butoxy ethanol + 40 ml perchloric acid (60%) using a LectroPol-5 (Struers) with a voltage of ~ 20–22 V (i.e., 0.35–0.40 A) for 45 s. The EBSD measurements were carried out with the Oxford EBSD camera attached to the JEOL, JSM-7900F FE-SEM at accelerate voltage of ~ 15 kV. A map size of 120 × 90 μm^2^ and a step size of 0.1 μm were applied in all the measurements.

## Results and discussion

**Figure 2:**
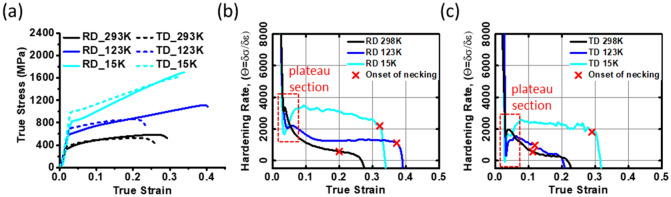
(**a**) Macroscopic true stress–strain curves and strain-hardening curves obtained from the tensile tests at 15–298 K with respect to (**b**) RD and (**c**) TD loading directions.

Figure 2a shows the macroscopic true stress–strain curves obtained from the tensile tests at 15–298 K. The strain-hardening rate (Θ = δσ/δε) was calculated from the true stress–strain curves, and the onset of necking (red X-marks) was predicted by the Considere's criterion^41^, as shown in Fig. 2b, c. The hardening rate increased dramatically with decreasing temperature, and it was relatively higher in RD than TD samples. The onset of necking is retarded with the increase of strain-hardening capacity. The total elongation is higher at 123 K and 15 K than that at 298 K, except for the result of TD sample at 123 K. In general, the strain-hardening contributes to the resistance to plastic instability (i.e., necking) during tensile deformation, thereby improving the ductility of materials^42,43^. The experimental results reveal that the necking resistance of CP-Ti is enhanced at cryogenic temperature. It is also noteworthy that three-stage hardening behaviour—(i) stage A: a decrease of hardening rate after yielding, (ii) stage B: an increase of hardening rate and (ii) stage C: a decrease of hardening rate in plastic flow regime—is observed in both RD and TD samples (see Fig. 2b, c), and the strain-hardening plateau between the stage A and B is further manifested as temperature decreases.

**Figure 3:**
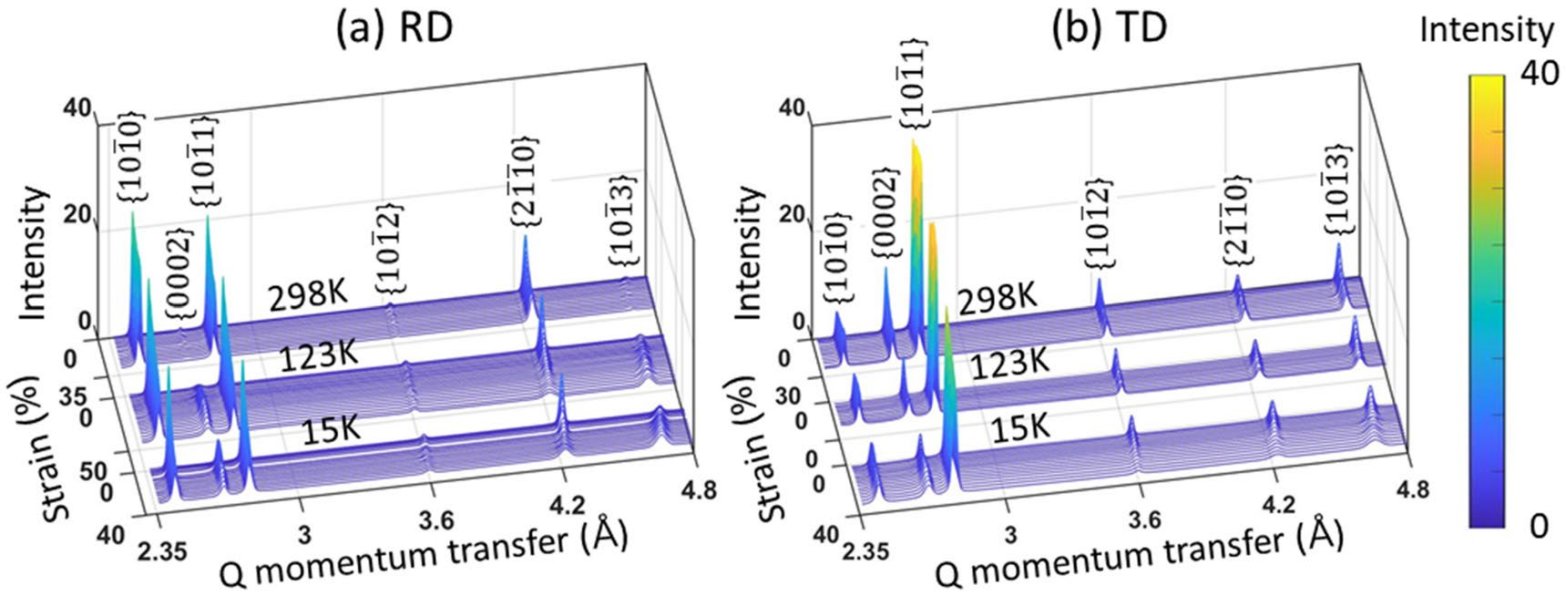
Neutron diffraction patterns collected during in-situ tensile tests at 15–298 K with respect to (**a**) RD and (**b**) TD loading directions. The lattice planes normal to the tensile loading direction are denoted to the {hkil} diffraction reflections.

**Figure 4:**
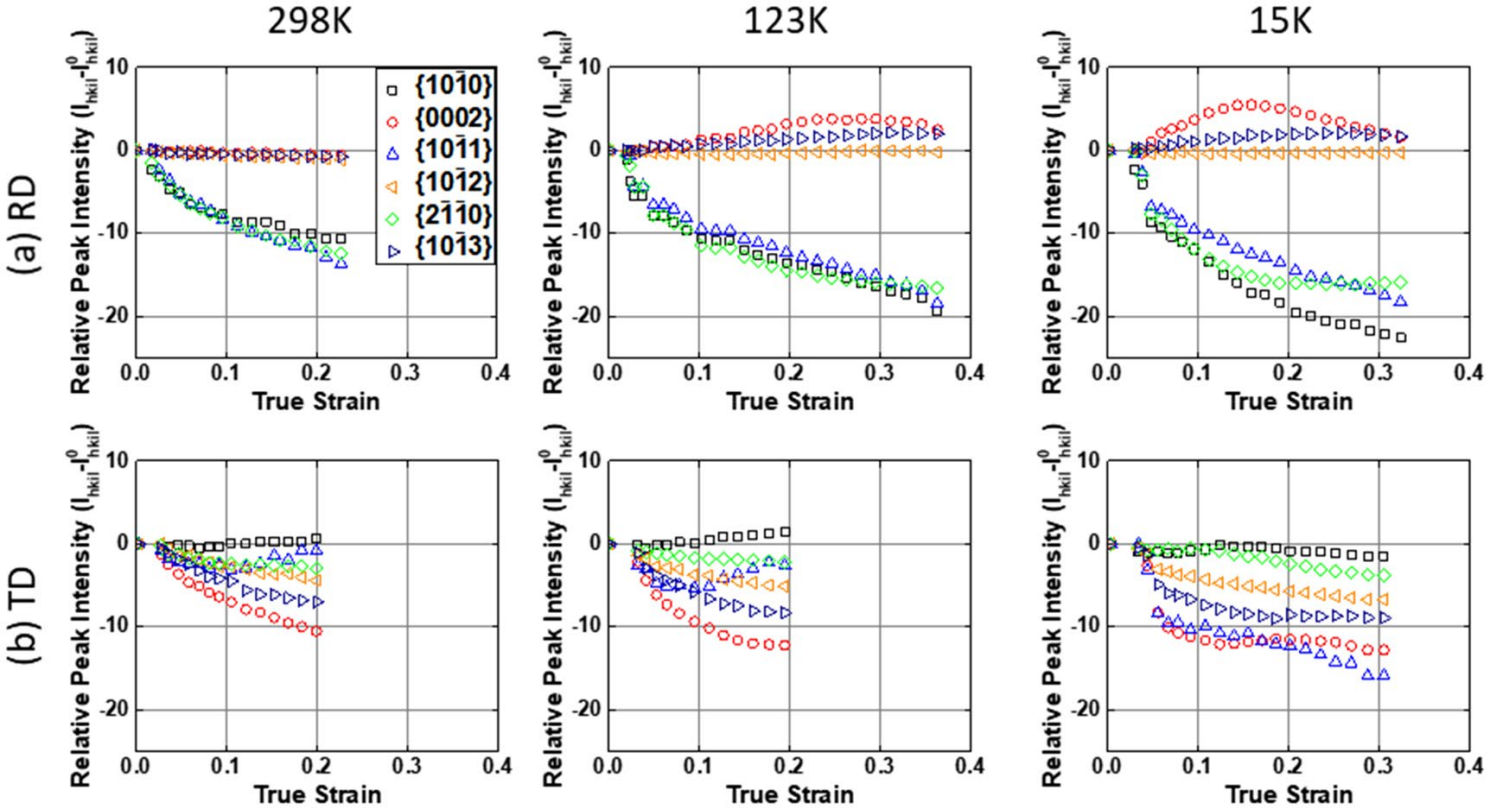
Evolution of relative diffraction peak intensities (i.e., $I_{hkil}$-$I_{hkil}^{0}$, where the $I_{hkil}$ is the {hkil} peak intensity at imposed strain and $I_{hkil}^{0}$ is the initial peak intensity) as a function of imposed strain at 298 K, 123 K and 15 K for (**a**) RD and (**b**) TD loading directions.

The neutron diffraction patterns collected during *in-situ* tensile tests are shown in Fig. 3, as a function of imposed strains. The {hkil} diffraction reflections correspond to the α-Ti lattice planes normal to the tensile loading direction. In Fig. 4, all the diffraction peak intensities (${\text{I}}_{{{\text{hkil}}}}$) were subtracted with their initial values (${\text{I}}_{{{\text{hkil}}}}^{0}$) at the beginning of tensile tests. Because the different initial textures were determined with respect to the loading directions in the strong crystallographic texture of rolled CP-Ti plate (see Fig. 1), the diffraction patterns are substantially different between the RD and TD samples. In the RD sample (see Fig. 3a), only the {$10\overline{1}0$}, {$10\overline{1}1$} and {$2\overline{1}\overline{1}0$} peaks are observed in the initial diffraction patterns. Note that the {$0002$} and {$10\overline{1}3$} peaks emerge during deformation at 123 K and 15 K. The {$0002$} and {$10\overline{1}3$} peak intensities initially increase as deformation progresses, and subsequently only for the {$0002$} peak intensity begin to decrease around the strains of 0.25 and 0.15 at 123 K and 15 K, respectively (see Fig. 4). At the same strain and temperature, the {$2\overline{1}\overline{1}0$} peak intensity tends to be convergent together with the decrease of {$0002$} peak intensity, and this convergent trend becomes more explicit at 15 K than 123 K.

**Figure 5:**
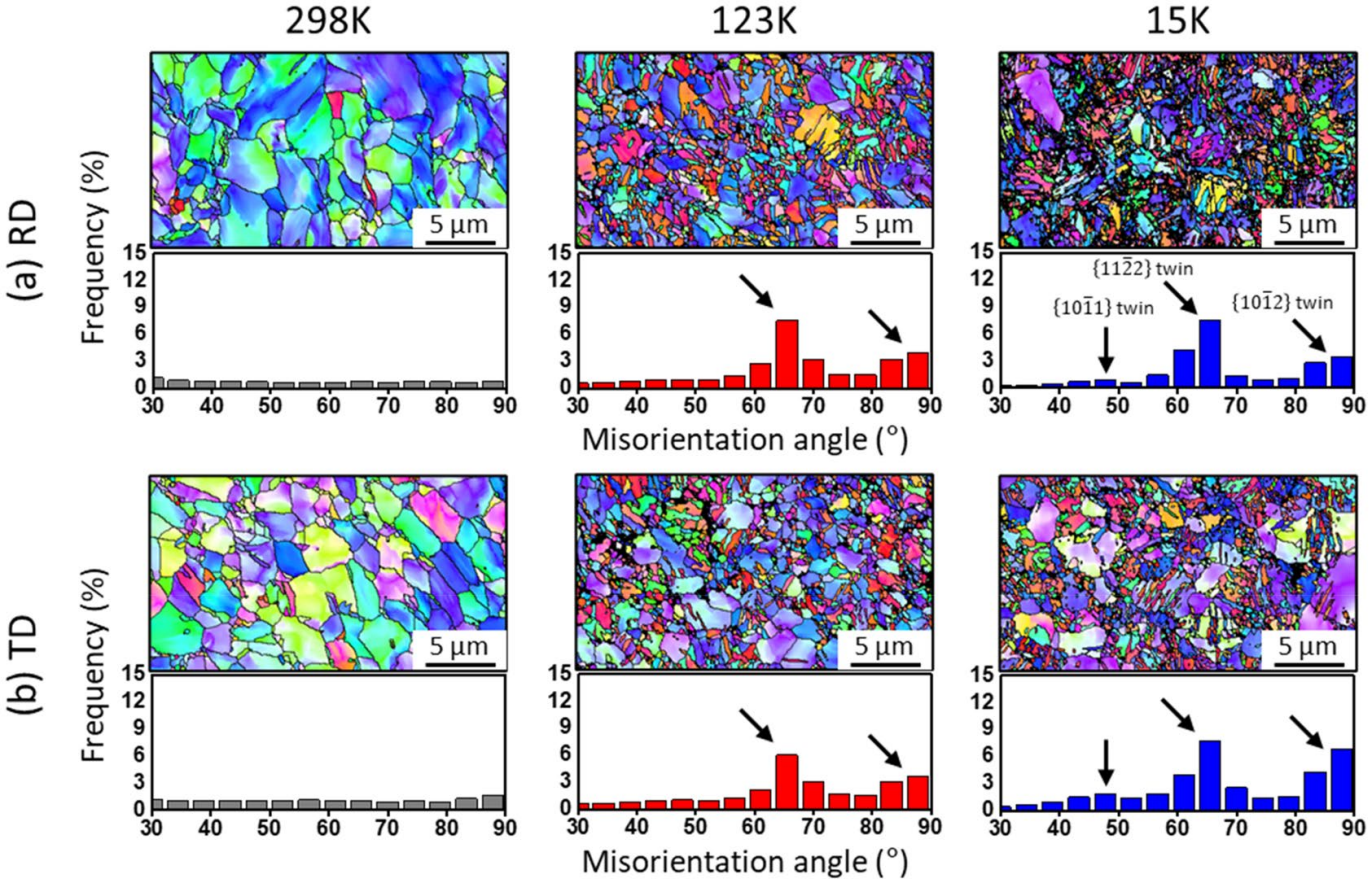
Distribution of grain boundary misorientation angles on the cross-sectional area below ~ 1 mm from the fracture surface of samples deformed at 298 K, 123 K and 15 K and the EBSD IPF maps with respect to the (**a**) RD and (**b**) TD loading directions.

The twinning-induced lattice reorientation results in the formation of new grain structures (i.e., twin daughter) within the original parent grains. The twin daughters consume their parent grains, and lead to the change of grain orientations. A certain diffraction peak intensity can be increased or convergent due to the twinning-induced lattice reorientation^30,31,33^. In this respect, the evolution of {$0002$}, {$10\overline{1}3$} and {$2\overline{1}\overline{1}0$} peak intensities is most likely related to the temperature-dependent twinning behaviour. To investigate the effect of temperature on twinning behaviour, the distribution of grain boundary misorientation angles on the surface of fractured samples was analysed by EBSD technique, as shown in Fig. 5. In the RD sample (see Fig. 5a), there is no strong misorientation peak at 298 K, whilst two strong misorientation peaks at ~ 64° and ~ 85° are found at 123 K and 15 K and one weak peak at ~ 50° in the deformation at 15 K. The twinning-induced grain refinement is more significant at lower temperature, as shown in the EBSD IPF maps. The misorientation angles of 64° and 85° are well-consistent with that of {$11\overline{2}2$} contraction and {$10\overline{1}2$} extensions twinning of α-Ti^44,45^, respectively. This indicates that the increase of {$0002$} and {$10\overline{1}3$} peak intensities is due to the formation of {$11\overline{2}2$} and {$10\overline{1}2$} twins. As for the {$10\overline{1}2$} and {$11\overline{2}2$} twinning, one of these two twinning systems rotates the lattice orientation to be favourable for the other twinning system, by which the {$10\overline{1}2$}-{$11\overline{2}2$} double twinning can be occurred in CP-Ti^46^. The initial rise and subsequent drop of {$0002$} peak intensity and the convergence of {$2\overline{1}\overline{1}0$} peak intensity (see Fig. 4) might be due to the {$10\overline{1}2$}-{$11\overline{2}2$} double twinning. The change in intensity evolution of these two peaks is more significant at 15 K than 123 K, suggesting that the occurrence of double twinning is more frequent at lower temperature.

In the TD sample (see Fig. 3), the {$10\overline{1}0$}, {$0002$}, {$10\overline{1}1$}, {$10\overline{1}2$}, {$2\overline{1}\overline{1}0$} and {$10\overline{1}3$} diffraction peaks were detected in the initial diffraction patterns. Figure 4b shows a rise in {$10\overline{1}0$}, a drop and rise in {$10\overline{1}1$} and a drop (298 K) or convergence (123 K) in {$0002$} peak intensities. Meanwhile, the evolution of forementioned three peak intensities significantly differ at 15 K, showing the multi-stage including two or more deflection points. The {$2\overline{1}\overline{1}0$} and {$10\overline{1}3$} peaks also exhibit a deflection and convergent point in the intensity evolutions at 15 K, unlike that at 298 K and 123 K. The variation of peak intensities is quite complex in the TD sample at 15 K compared to that of the RD sample. In particular, the multi-stage in peak intensity evolution plausibly indicates the tertiary twinning and/or next sequence of twinning. The misorientation peaks at 64° and 85° demonstrate that the {$10\overline{1}2$} and {$11\overline{2}2$} twinning are predominant in the TD sample at cryogenic temperature (see Fig. 5b). At 15 K, for the misorientation peak at 50°, this angle is quite similar to the reorientation angle (54°) of {$10\overline{1}1$} contraction twinning^44^, and its peak value is higher in the TD than RD sample. The activation of various twinning systems results in the texture evolution^7,13^, depending on the initial crystal orientations. In practice, it has been reported that the activated twinning system was affected by the temperature and initial texture of pure Ti during rolling process, and the different textures were developed due to the activated twinning system and twinning sequences depending on orientations^47^. For this respect, the evolution of peak intensity reveals the more complex twinning behaviour in the TD sample at 15 K, suggesting that a distinct texture of Ti can be developed during deformation at extremely low temperature. Note that the fraction of twins is intuitively seen to be higher at lower temperature, and more in the RD than TD samples (see the IPF maps of Fig. 5). The twinning-induced hardening effect is likely higher in the RD than TD samples, and it increases with decreasing temperature.

**Figure 6:**
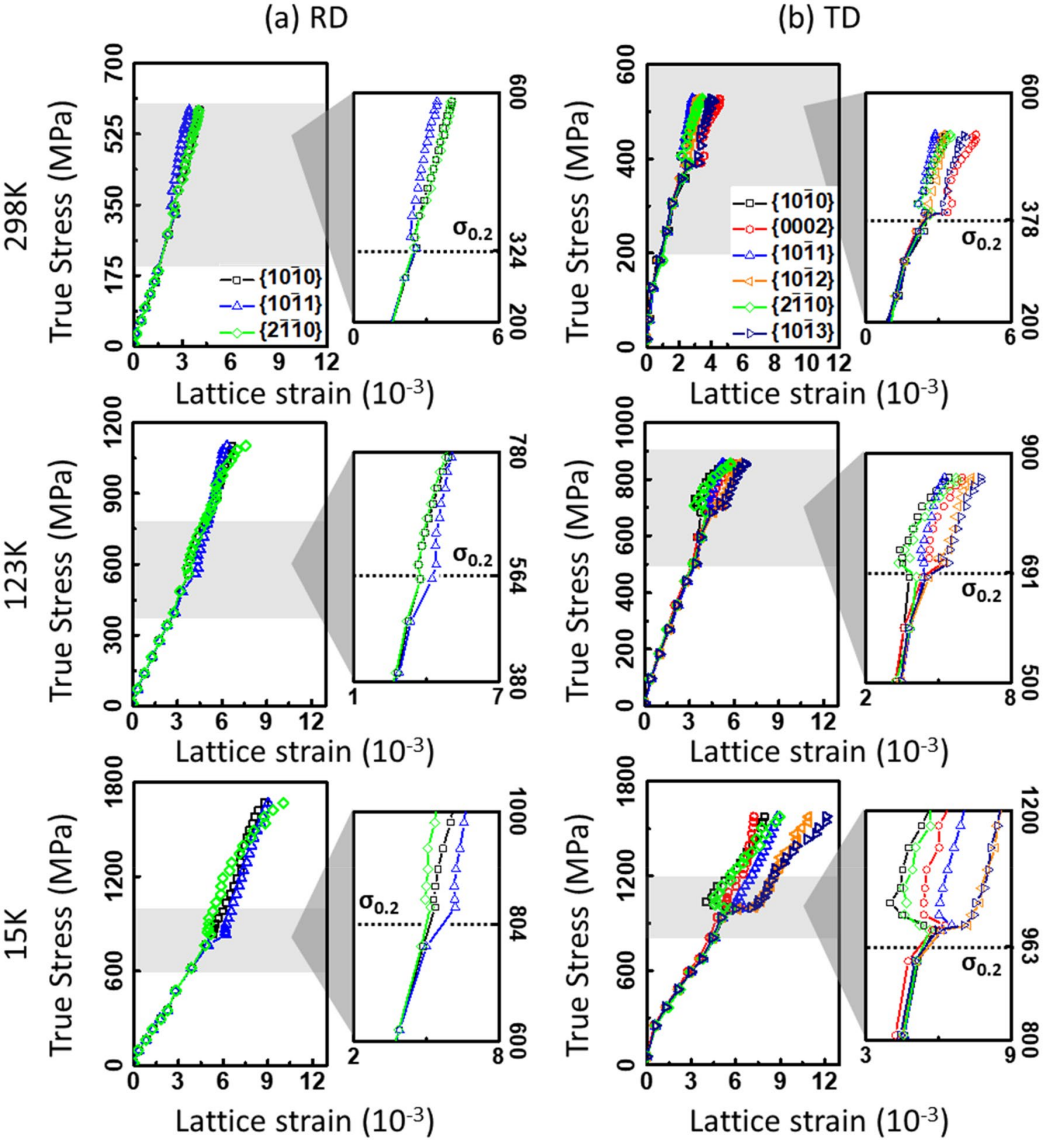
Evolution of lattice strains as a function of applied stress at 15–298 K for (**a**) RD and (**b**) TD loading directions. The sections of microyielding in {hkil} grain families (grey-region) are magnified, and the same axial ranges are given to these magnified graphs with 6 micro strains in x-axes and 400 MPa in y-axes. The data deviation is quite small less than 0.3 (not shown in the figure).

The evolutions of lattice strains in {hkil} grain families are shown in Fig. 6, as a function of the macroscopic true stress. In the elastic regime, the lattice strains from various {hkil} planes are generally equivalent at all the temperature conditions (see subfigure of Fig. 6), indicating that the elastic anisotropy of current sample is not significant. The deviation from linearity of the lattice strains in {hkil} planes occurred at macroscopic yielding point. This signifies that the stress, which is constantly held in {hkil} planes, is anisotropically redistributed by the plastic deformation, depending on the crystal plane. The non-linear lattice strain response of polycrystalline α-Ti is closely related to the orientation-dependent slip mechanism^30–33^. In this regard, certain grains oriented to favour a particular slip system with low CRSS are preferentially yielded with the initiation of dislocation slip, and then the load shed from the yielded grains would be redistributed to the other grains oriented to favour the slip systems with higher CRSS, for that reason, these grains remain elastic. The former and later grains can be denoted to plastically ‘soft’ and ‘hard’ grains, respectively. As a result, the hard grains are further elastically-deformed with carrying the shed load, which in turn, results in the rapid increase of lattice strain due to the high applied load^31,32^ (see Fig. 6).

**Figure 7:**
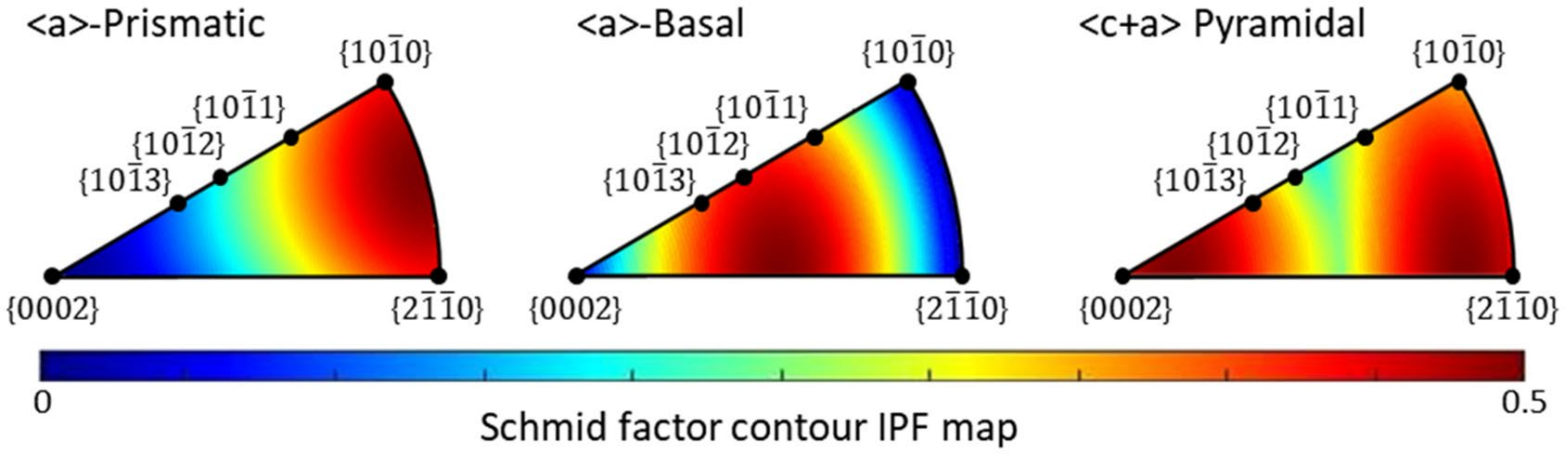
IPF of maximum Schmid factor (generated by the toolbox MTEX developed in MATLAB^TM50^) for < a > -prismatic (Pri < a >) and -basal (Ba < a >), and < c + a > -pyramidal (Pyr < c + a >) slip systems of α-Ti.

Two intrinsic material factors, Schmid factor and CRSS, are used to assess the activation of certain slip systems^6^. Thus to elucidate the anisotropic lattice strains in {hkil} grain families, the IPFs of maximum Schmid factor were plotted for the common three slip systems of α-Ti, that is, *Pri* < a > , *Ba* < a > and *Pyr* < c + a > slip systems^23,48,49^, as shown in Fig. 7. The texture components corresponding to the crystal lattice planes normal to the tensile axis were marked on the IPFs.

The {$10\overline{1}1$} grain family has equivalent Schmid factor value (0.35) for all the three slip systems (see Fig. 7), nevertheless, it is expected that the *Pri* < a > slip was preferentially activated in this grain families due to the lower CRSS^22^ than the other two slip systems. The {$10\overline{1}0$} and {$2\overline{1}\overline{1}0$} grain families have high Schmid factor (0.45) for *Pri* < a > slip, as such these two grain families are likely to favour the *Pri* < a > slip together with the {$10\overline{1}1$} grain family. At 298 K, the {$10\overline{1}0$}, {$2\overline{1}\overline{1}0$} and {$10\overline{1}1$} grain families are mainly existent in the RD sample and retained the linearity of the lattice strains with respect to the applied stress even in the plastic regime (see Fig. 6a). The linear lattice strain response indicates that the load assigned to each grain family is not significantly redistributed after the yielding of grains, leading to the homogeneous deformation. This is attributed to the dominant activation of *Pri* < a > slip systems over the other two slip systems due to its high Schmid factor for all the three grain families. In Fig. 2b, the three-stage hardening behaviour is nearly indistinct in the RD sample at 298 K, showing the monotonic strain-hardening curve compared to the other experimental cases. There are reported two main reasons of three-stage hardening behaviour in CP-Ti; twinning-induced hardening mechanism^51^ or dislocation-based plasticity^24,25,32^. In our previous study^6^, it has been found that only small amount of twins (below ~ 5%) were formed in the stage B-C section of the present material during tensile testing at both room and cryogenic temperature. Therefore, the monotonic strain-hardening behaviour is ascribed to the homogeneous deformation dominated by the *Pri* < a > slip system. However, admittedly, the lack of diffraction peaks from the other crystal planes limits the full understanding of the microplasticity of α-titanium.

In addition to the {$10\overline{1}0$}, {$10\overline{1}1$} and {$2\overline{1}\overline{1}0$} grain families, the lattice strains in the {$10\overline{1}2$}, {$10\overline{1}3$} and {$0002$} grain families were also analysed in the TD samples (see Fig. 6b). According to the Schmid factor analysis (see Fig. 7), the later three grain families have relatively low Schmid factor (0–0.2) for *Pri* < a > slip in comparison with the formers. In the {$10\overline{1}2$} grain family, the Schmid factor for *Ba* < a > slip is as high as 0.43, but that for *Pyr* < c + a > slip is not as high. Besides, the {$0002$} grain family has the highest Schmid factor (0.5) for *Pyr* < c + a > slip. Likewise to the RD sample, the {$10\overline{1}0$), {$10\overline{1}1$} and {$2\overline{1}\overline{1}0$} grain families are microplastically deformed due to the high Schmid factor and the lowest CRSS for *Pri* < a > slip in the TD sample at 298 K, thereby shedding the load at yielding point (~ 378 MPa). At the same time, the {$0002$} grain family further carries the load shed from the soft grain families, and subsequently this grain family shed the load at ~ 406 MPa plausibly due to the initiation of hard *Pyr* < c + a > slip, showing the deviations from linearity of the lattice strain. The {$10\overline{1}2$} grain family is favourable for the activation of *Ba* < a > slip, for which this grain family less shed and carries the load relative to the other grain families, resulting in the linear lattice strain response. The {$10\overline{1}3$} grain family exhibits the lattice strain evolution similar to that of the {$0002$} grain family, suggesting that the *Pyr* < c + a > slip acts primarily as a carrier of microplasticity in both grain families. Otherwise, the plastic deformation is likely to be accommodated by the *Ba* < a > slip in the {$10\overline{1}3$} grain family, in which the activation of *Ba* < a > slip is likely to be harder than that in the {$10\overline{1}2$} grain family due to the unfavourable crystallographic condition; the Schmid factor for *Ba* < a > slip is 0.43 in {$10\overline{1}2$} and 0.4 in {$10\overline{1}3$} grain families. The activity of slip systems could be rigorously quantified by the analysis via crystal plasticity simulations, however, it is beyond the scope of the current study.

In the TD sample at 298 K, the load-shedding and -carrying between soft and hard grain families results in the deviation from linearity of the lattice strains (see Fig. 6b), and the three-stage hardening behaviour (see Fig. 2c) is attributed to the heterogeneous microplasticity caused by the load-redistribution. Interestingly, as temperature decreases, the three-stage hardening behaviour becomes much severer with the increasing deviation of linearity in the lattice strains, and in particular for the RD samples, the change of strain-hardening curves from monotonic (298 K) to three-stage character (123 K and 15 K) implies a significant transition in the deformation mechanisms triggered by the temperature dependent microplasticity. At 123 K and 15 K, the lattice strains practically differ from that at 298 K. For the RD (see Fig. 6a), after the yielding points, the {$10\overline{1}1$} grain family shows the relatively rapid increase of lattice strain to the other two grain families, carrying more load and resulting in the non-linear lattice strain response. The deviation of linearity in the lattice strains is more significant at 15 K than 123 K. Likewise, for the TD (see Fig. 6b), the {$10\overline{1}1$} grain family less shed load at 123 K, and even carries more load that in the {$0002$} grain family at 15 K. It should be also noted that the {$10\overline{1}2$} grain family exhibits the higher load-carrying capacity at cryogenic temperatures at yielding points, and the {$0002$} grain family strongly shed load at ~ 708 MPa (123 K), and more significantly at 999 MPa (15 K) contrary to that at room temperature. The analysis of lattice strains reveals that the characteristics of grain families are largely changed by the cryogenic temperature, even though the crystal orientation conditions are same with that at room temperature. The plastically soft-hard transition of grain families is likely related to the change of CRSS for slip systems depending on the temperature. The prior studies reported that the CRSS value increased differently between *Pri* < a > and *Ba* < a > slip with decreasing temperature, and the details of Pyr < c + a > slip is still unclear^52,53^. This indicates that the CRSS ratio between the three slip systems can be changed by decreasing temperature, so that the preferred slip system in particular grain orientation might be altered at cryogenic temperature. In this respect, the {$10\overline{1}1$} grain family has the same Schmid factor (0.35) for all the three slip systems, for that reason, the active slip system is likely to be easily altered from *Pri* < a > to *Ba* < a > or *Pyr* < c + a > slip. Although the Schmid factor value is the lowest for the *Pri* < a > and *Ba* < a > , and the highest for *Pyr* < c + a > slip in the {$0002$} grain family, the load is strongly shed at cryogenic temperature (see Fig. 6b). The {$10\overline{1}2$} grain family with the high Schmid factor (0.43) for *Ba* < a > slip became harder than the {$0002$} grain family. It is thus expected that the activation of *Pyr* < c + a > slip might be easier at lower temperature. However, there is an uncertainty of the CRSS for slip systems of α-Ti in low temperature range, because the CRSS values were only estimated by the macroscopic experiments using the bulk samples with a microstructural uncertainty in the last few decades^27–29^. Accordingly, the next step is to evaluate the CRSS for slip systems of α-Ti with an appropriate experimental technique^54^ at cryogenic temperature.

**Figure 8:**
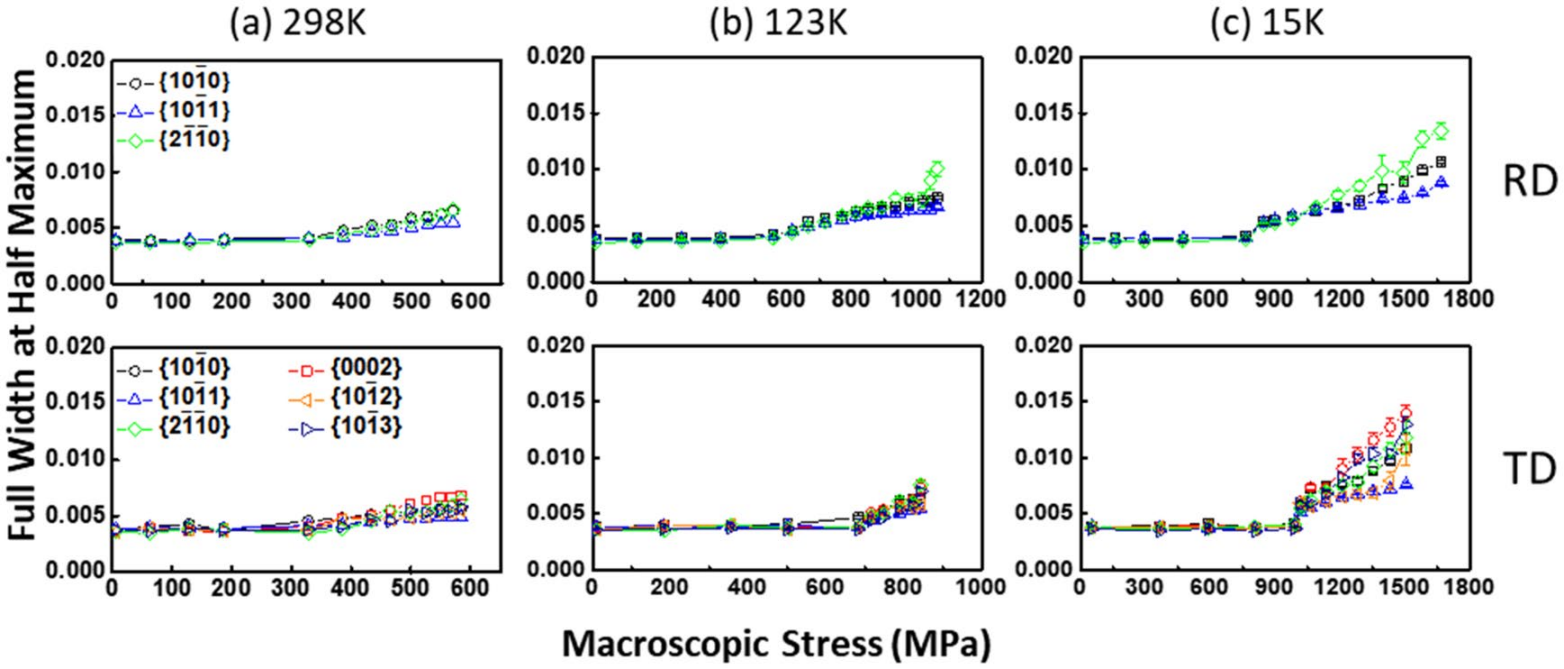
The relative half width of {hkil} diffraction peaks (i.e., FWHMs divided by its peak positions) analysed as a function of applied stress at (**a**) 298 K, (**b**) 123 K and (**c**) 15 K for RD and TD loading conditions.

Since the heterogeneous defect structure such as dislocations and stacking faults broaden the peak width whilst materials are strain hardened, the neutron diffraction line broadening quantified by the normalised full width at half maximum (FWHM) was used to estimate the accumulated dislocation density, as shown in Fig. 8. The FWHMs increases in the plastic region, and more significantly at cryogenic temperature. This is likely due to the higher dislocation density at lower temperature, because the dynamic recovery is suppressed with decreasing temperature, which in turn facilitates the dislocation accumulation^55^. Hence, the dislocation-based mechanisms contribute to the high strain-hardening capacity and ductility at cryogenic temperature (see Fig. 2) together with the twinning-induced hardening mechanism. The previous studies reported that the uniform elongation of CP-Ti is highly anisotropic at room temperature, showing the inferior ductility along the TD loading direction^6,7,25^. The twinning is absent in both RD and TD samples at 298 K (see Fig. 5). This indicates that the effect of orientation dependent dislocation slip is significant on the strain-hardening behaviour. However, in the TD (see Fig. 2c), the tensile properties (especially uniform elongation) are greatly improved at 15 K. The FWHMs are equally increased during plastic deformation at 298 K and 123 K, whilst that of grain families are inequivalent at 15 K (see Fig. 8), in which the FWHM value is the lowest in the {$10\overline{1}1$} grain family (RD/TD) and the highest in the {$0002$} grain family (TD). This result is correlated with the temperature dependent lattice strain response (see Fig. 6). The generation of dislocations, which could be promoted at cryogenic temperature due to the change of CRSS ratio between slip systems, is anticipated to give rise to the strain-hardening capacity under various grain orientations along the TD loading direction.

## Conclusion

In this study, the anisotropic lattice behaviour of Grade 2 CP-Ti was investigated by using *in-situ* neutron diffraction test in tension at temperatures of 15–298 K. As the deformation temperature decreases, the ductility was improved with the increase of strain-hardening capacity. At room temperature, the monotonic and three-stage characteristics were exhibited in the strain-hardening curves of the RD and TD samples, respectively. The three-stage character was also observed in the RD sample at cryogenic temperature, and the strain-hardening plateau was more significant in both RD and TD samples with decreasing temperature. The RD grain families, which are oriented to favour the *Pri* < a > slip, showed the linear lattice strain response. In contrast, the non-linear lattice strain response was shown in the TD grain families capable of activating the *Ba* < a > and *Pyr* < c + a > slip in addition to the *Pri* < a > slip. The three-stage hardening behaviour was attributed to the activation of *Ba* < a > or *Pyr* < c + a > slip in the relatively hard grains that results in the load-redistribution. At cryogenic temperature, the RD grain families also showed the non-linear lattice strain response, and the deviation of linearity in the lattice strains became more significant with decreasing temperature. The plastically soft-hard transition occurred in several grain families at cryogenic temperature, leading to the deviation of linearity in the lattice strains. The lattice strain results suggest that the CRSS ratio between the slip systems of α-Ti could be changed at cryogenic temperature, possibly promoting the activation of *Pyr* < c + a > slip system.

The deformation twinning was more active at lower deformation temperature. The neutron diffraction peak intensity revealed the complex twinning behaviour resulting from the multiple twinning activity at extremely low temperature (15 K). The suppressed dynamic recovery and twinning-induced hardening give rise to the strain-hardening capacity at cryogenic temperature. Meanwhile, the temperature dependent lattice strain response is anticipated to be involved in the strain-hardening behaviour. The findings in the present study provide a comprehensive understanding of deformation behaviour in α-Ti at cryogenic temperature.

## Supplementary Information

Supplementary Information.
