# Supplementary material for: In-situ neutron diffraction study of lattice deformation behaviour of commercially pure titanium at cryogenic temperature
Source: Sci Rep. 2022 Mar 8;12:3719. doi: 10.1038/s41598-022-07640-3 (PMC8904482; doi:10.1038/s41598-022-07640-3)
Supplement: Supplementary file 1 — Supplementary Information. [file 41598_2022_7640_MOESM1_ESM.docx]

SUPPLEMENTARY MATERIAL

***In-situ* neutron diffraction study of lattice deformation behaviour of commercially pure titanium at cryogenic temperature**

Min-Su Lee^1^, Takuro Kawasaki^2^, Takayuki Yamashita^2^, Stefanus Harjo^2^, Yong-Taek Hyun^3^, Youngung Jeong^4^_,_ Tea-Sung Jun^1,5*^

^1^Department of Mechanical Engineering, Incheon National University, Incheon 22012, Republic of Korea

^2^J-PARC Center, Japan Atomic Energy Agency, Tokai, Ibaraki 319-1195, Japan

^3^Metallic Materials Division, Korea Institute of Materials Science, Changwon 51508, Republic of Korea

^4^School of Materials Science and Engineering, Changwon National University, Changwon, Republic of Korea

^5^Research Institute for Engineering and Technology, Incheon National University, Incheon 22012, Republic of Korea

**1. Diffraction patterns with scattering vector (Q_⊥_)**

The diffracted neutron beams were collected by two diffraction detectors as denoted north and south banks. The former and latter detector acquired the diffraction beams with scattering vector of Q*_∥_* and Q_⊥_, respectively. Each neutron diffraction beams reflects the lattice planes normal to the tensile loading direction (in Q*_∥_*) and normal direction in rolled plane of CP-Ti plate (in Q_⊥_). The neutron diffraction patterns measured by the south bank (Q_⊥_) are shown in Fig. S1, as a function of imposed strain. Because the RD and TD tensile samples have the same plane normal to the rolled plane, the diffraction patterns are quite similar between RD and TD sample.


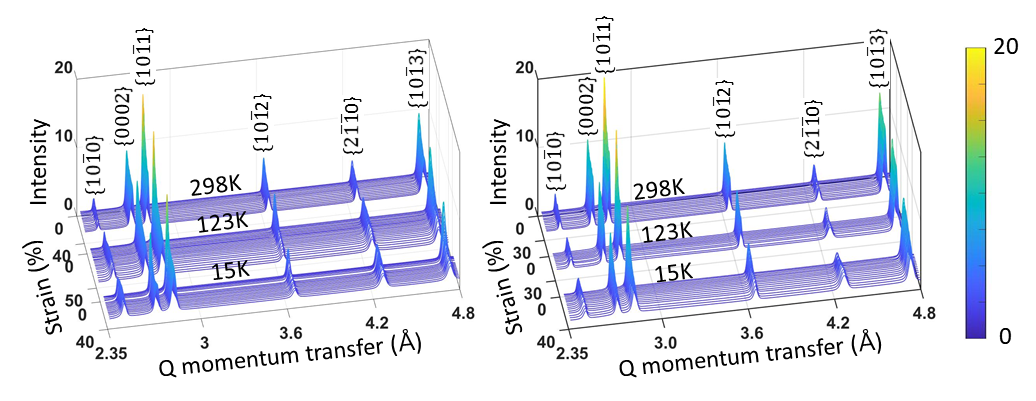


*Fig. S1. Diffraction patterns collected at the detector bank with scattering vector of Q_⊥_ as a function of imposed strain at 15-298K for RD and TD loading conditions.*

**2. Data reliability**

Fig. S2 shows the measured diffraction patterns with the scattering vector of Q*_∥_* at immediately before the fracture of RD and TD samples at 298 K, 123 K and 15 K. Full pattern fitting of the diffraction patterns was conducted by Rietveld refinement using the Z-Rietveld software. In Fig. S2, the measured and fitted data were highlighted by the black dots and red lines, respectively. The difference between these two data (blue lines) can be seen at the bottom of the diffractograms. The diffraction peaks of α-titanium phase were well-fitted, but the several wrong fitting results were occurred in the Rietveld refinement. In this regard, the extensometer was used during the tensile testing, and the parts of extensometer were made of aluminium. It is thought that the aluminium parts were involved in the beam diffraction due to the alignment issue and etc, leading to the wrong fitting results in the Rietveld refinement for titanium crystal structure. In practice, the wrong fitted diffraction peaks were revealed as fcc aluminium phases through the Rietveld refinement for aluminium crystal structure. Except for the aluminium phases, the fitted data is greatly coincident with the measured data, even though the diffraction patterns were taken from the data obtained at severe plastic deformation.

*
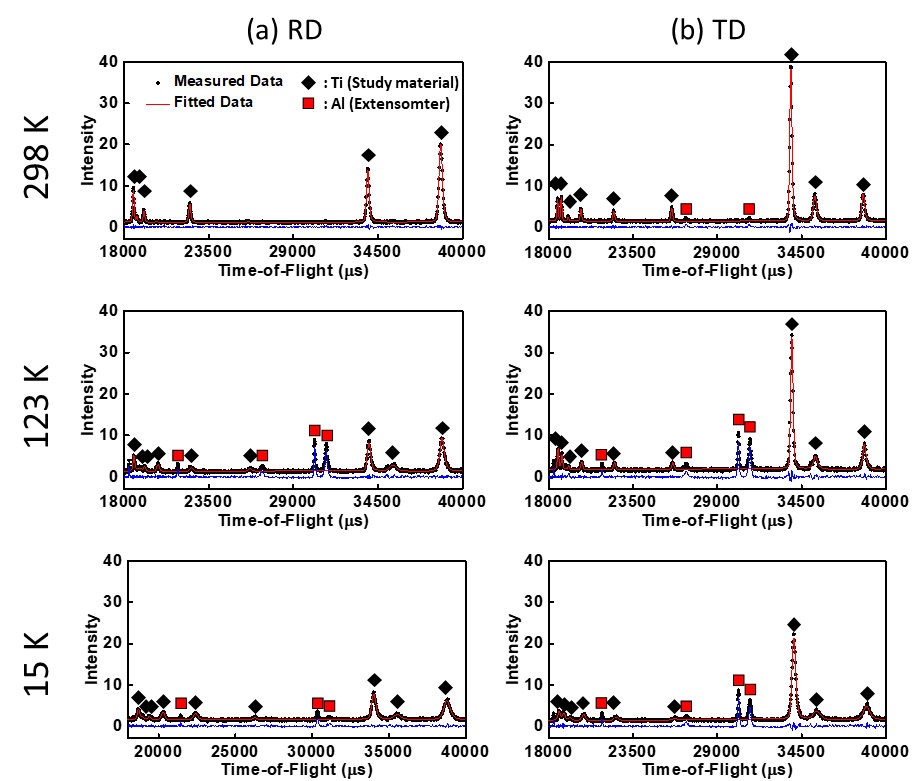
*

*Fig. S2. The measured neutron diffraction patterns (black dot) with scattering vector of Q_∥_ and diffractograms fitted by Rietveld refinement (red line) at immediately before the fracture of (a) RD and (b) TD samples under 298 K, 123 K and 15 K. The difference between measured and fitted data is highlighted by blue-coloured lines. Note that the diffraction peaks of red square is correspond to the aluminium phase resulting from the strain gauge attached on the tensile sample, and the black one is correspond to the α-titanium phase for the present study material.*
